# BDMediLeaves: A leaf images dataset for Bangladeshi medicinal plants identification

**DOI:** 10.1016/j.dib.2023.109488

**Published:** 2023-08-11

**Authors:** Saiful Islam, Md. Rayhan Ahmed, Siful Islam, Md Mahfuzul Alam Rishad, Sayem Ahmed, Toyabur Rahman Utshow, Minhajul Islam Siam

**Affiliations:** Department of Computer Science and Engineering, United International University, United City, Madani Avenue, Badda, Dhaka, 1212, Bangladesh

**Keywords:** Medicinal leaf classification, Computer vision, Deep learning, Image processing

## Abstract

This paper introduces a newly curated dataset named “BDMediLeaves” that includes a diverse collection of leaf images of ten distinct medicinal plants from various regions in Dhaka, Bangladesh. The ten distinct categories are Phyllanthus emblica, Terminalia arjuna, Kalanchoe pinnata, Centella asiatica, Justicia adhatoda, Mikania micrantha, Azadirachta indica, Hibiscus rosa-sinensis, Ocimum tenuiflorum, and Calotropis gigantea. The dataset contains a total of 2,029 original leaf images, along with an additional 38,606 augmented images. Each original image was meticulously captured under natural lighting conditions with an appropriate background. Experts provided accurate labeling for each image, ensuring its seamless integration into various machine learning (ML) and deep learning (DL) models. This comprehensive dataset holds immense potential for researchers in utilizing various ML and DL methods to make significant advancements in the healthcare and pharmaceutical sectors. It serves as a valuable resource for future investigations, laying the foundation for crucial developments in these domains.

Specifications Table

**Table:** 

Subject	Computer Sciences, Agricultural Sciences
Specific subject area	Computer Vision, Image processing, Image classification
Data format	Two-dimensional (2D) RGB images. Both the original raw digital images and the augmented images are in (.jpg) format.
Type of data	Image
Data collection	Over the range of six months from December 2022 to May 2023, we gathered quality images of healthy leaves with natural backgrounds from various locations within Dhaka City, using the camera of an iPhone 13. Subsequently, the images were resized to 3024 × 4032 pixels, and any low-resolution images were removed. As a result, we obtained a total of 2,029 leaf images distributed among ten classes, with each class containing approximately 200 images except the Ocimum tenuiflorum class which contains 151 images. The dataset also provides 38,606 augmented images. Throughout the process, we ensured improved luminosity, and a well-distributed light source, and captured the images without any noticeable shadows (nearly 0% shadow capture).
Data source location	There are five locations from where we collected images - 1. The National Botanical Garden of Bangladesh, Mirpur, Dhaka. 2. United International University playground in Dhaka. 3. Avi Garden Nursery in 100 feet road, Vatara, Dhaka. 4. Green Garden Nursery in 100 feet road, Vatara, Dhaka. 5. Barisal Nursery in Savar, Dhaka. **City/Town/Region**: Dhaka **Country**: Bangladesh
Data accessibility	Repository: Mendeley Data DOI: 10.17632/gk5x6k8xr5.1 URL: https://data.mendeley.com/datasets/gk5x6k8xr5
Related research article	Not Applicable

## 1 Value of the Data

• The proposed leaf image-based named “BDMediLeaves” dataset is valuable because it provides a large collection of leaf images from ten different medicinal plants that are frequently encountered in diverse regions of Bangladesh.
• The dataset presented herein provides an essential resource for researchers engaged in the identification of diverse medicinal plant species through the utilization of artificial intelligence and computer vision. It facilitates the training and evaluation of various ML and DL models for a wide variety of tasks, including object detection, image segmentation, classification [1,2], and the analysis of specific leaf characteristics such as shape, color, and texture.
• It can be re-used for a variety of tasks, including botanical research focused on studying plant morphology and leaf characteristics. Additionally, the dataset can be utilized for plant disease identification purposes too.
• The dataset possesses significant potential in academic environments, facilitating the instruction of plant identification, taxonomy, and the significance of medicinal plants.
• The dataset is open to the public, enabling researchers to employ it in their respective research activities.

## 2 Objective

Medicinal plants are recognized as a significant and plentiful source of bioactive compounds, such as alkaloids, terpenoids, flavonoids, and polyphenols, which are well-known for their therapeutic properties. These compounds possess the capacity to defend against pathogens, mitigate inflammation, treat skin conditions, facilitate wound healing, and enhance the immune system [3]. These are also deemed to be of significant importance in the progression of contemporary medical practices and the exploration of new pharmaceutical compounds. The purpose of the dataset is to provide a comprehensive collection of leaf images pertaining to ten distinct medicinal plant species that are frequently encountered in various regions of Bangladesh. These species include *Phyllanthus emblica, Terminalia arjuna, Kalanchoe pinnata, Centella asiatica, Justicia adhatoda, Mikania micrantha, Azadirachta indica, Hibiscus rosa-sinensis, Ocimum tenuiflorum,* and *Calotropis gigantea*. This dataset is intended to be used for training and evaluating ML and DL-based models for medicinal plants/leaves classification, focusing on the ten plant species mentioned earlier. It is noteworthy to acknowledge the present insufficiency of publicly accessible standardized datasets concerning medicinal leaves or plants found in Bangladesh. The dataset presented possesses significant potential as a valuable asset for researchers, scientists, and practitioners operating within diverse domains, with a particular emphasis on herbal medicine, plant-based remedies, medicinal plant identification, ML, DL, and computer vision research, where it can serve as a benchmark for image recognition algorithms and classification models. Furthermore, this dataset holds relevance for public awareness and education initiatives, as well as for fields extending beyond traditional medicine, including agriculture, environmental monitoring, and remote sensing.

## 3 Data Description

**Fig. 1:**
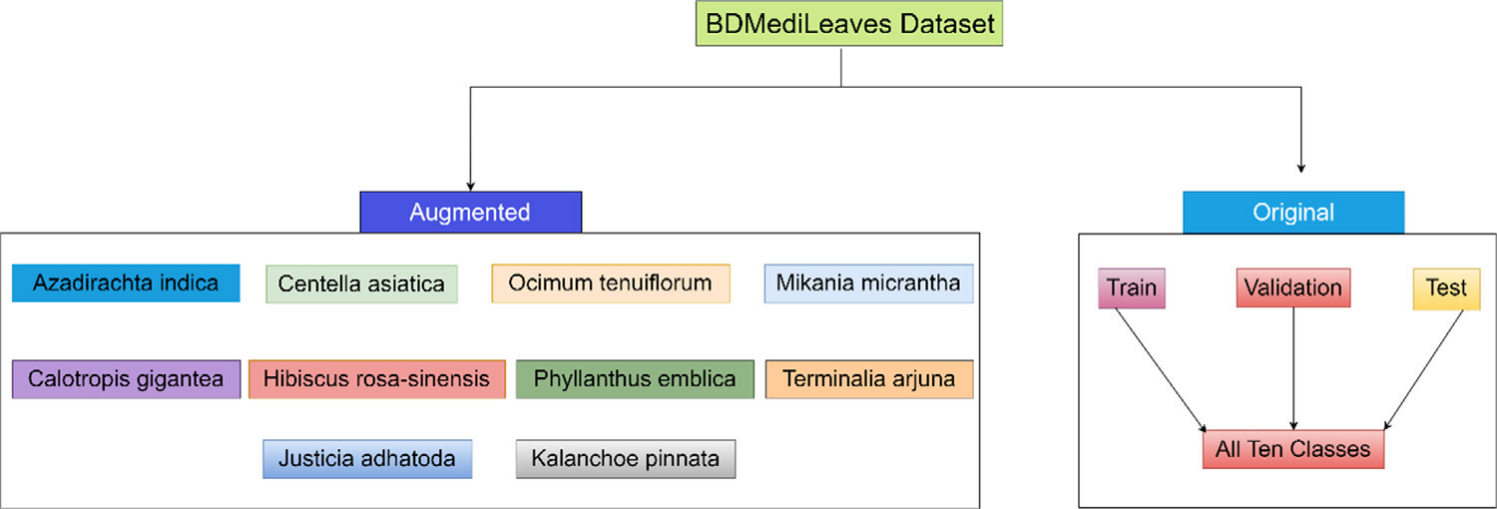
Directory tree of the proposed BDMediLeaves dataset.

The dataset named “BDMediLeaves” comprises a collection of images sourced from multiple nurseries and botanical gardens located in Dhaka, Bangladesh. The dataset comprises 2,029 images, which are arranged in the manner depicted in Fig. 1. It represents the directory tree of the proposed dataset. The primary directory is denoted as “BDMediLeaves” and encompasses two subdirectories, one for originally collected raw leaf images and the other for images that have undergone various image augmentation techniques. The original directory consists of three subdirectories: Train, Validation, and Test, with a proportional allocation of 70%, 20%, and 10% of images, respectively. All ten classes of images are present in each of the three subdirectories. In the augmented directory, we provide class-wise augmented images for researchers to use according to their needs. A total of 38,606 augmented images, each with a size of 512 × 512 pixels, are provided.

**Fig. 2:**
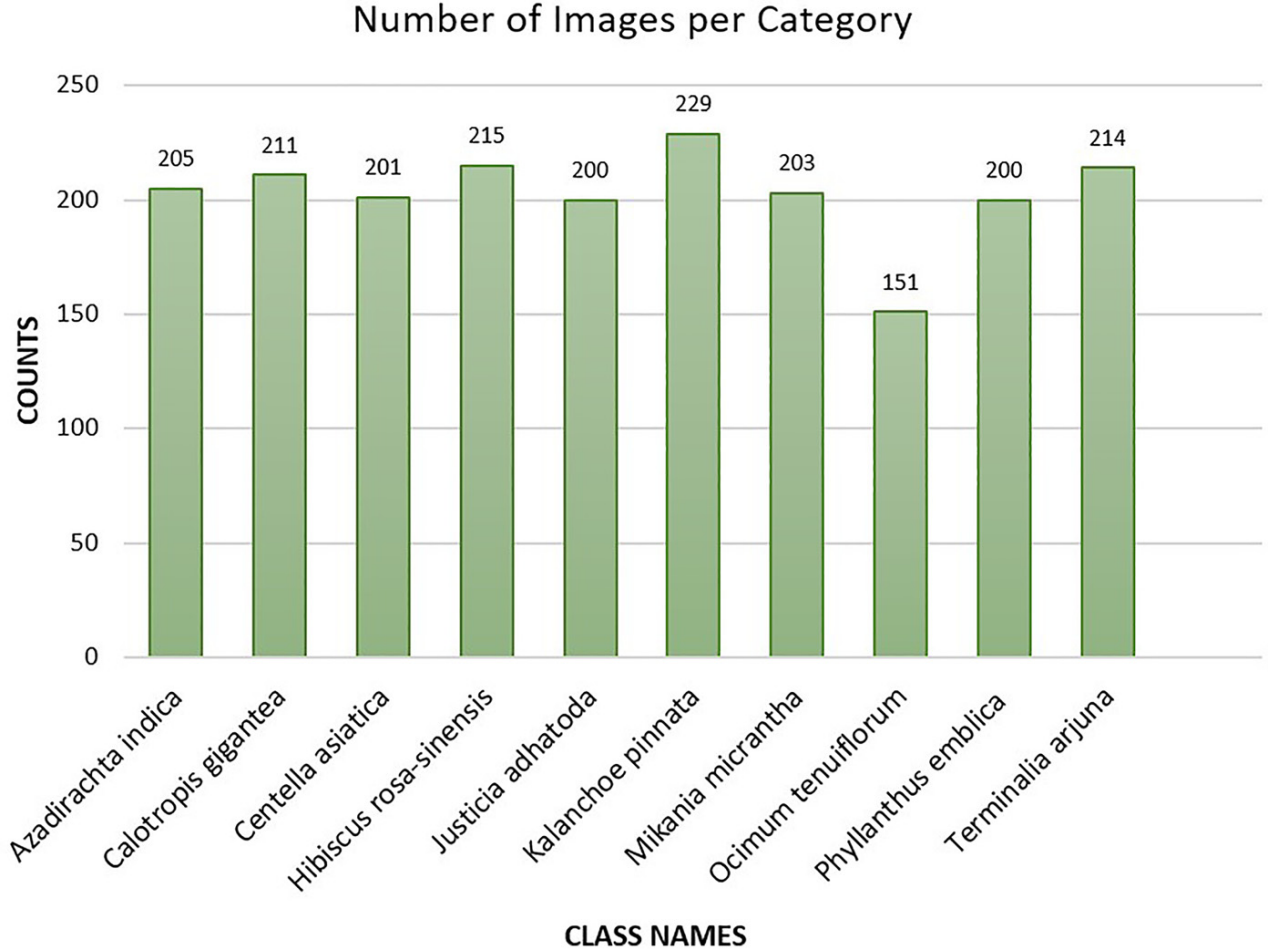
Class-wise image distribution in the BDMediLeaves dataset.

**Fig. 3:**
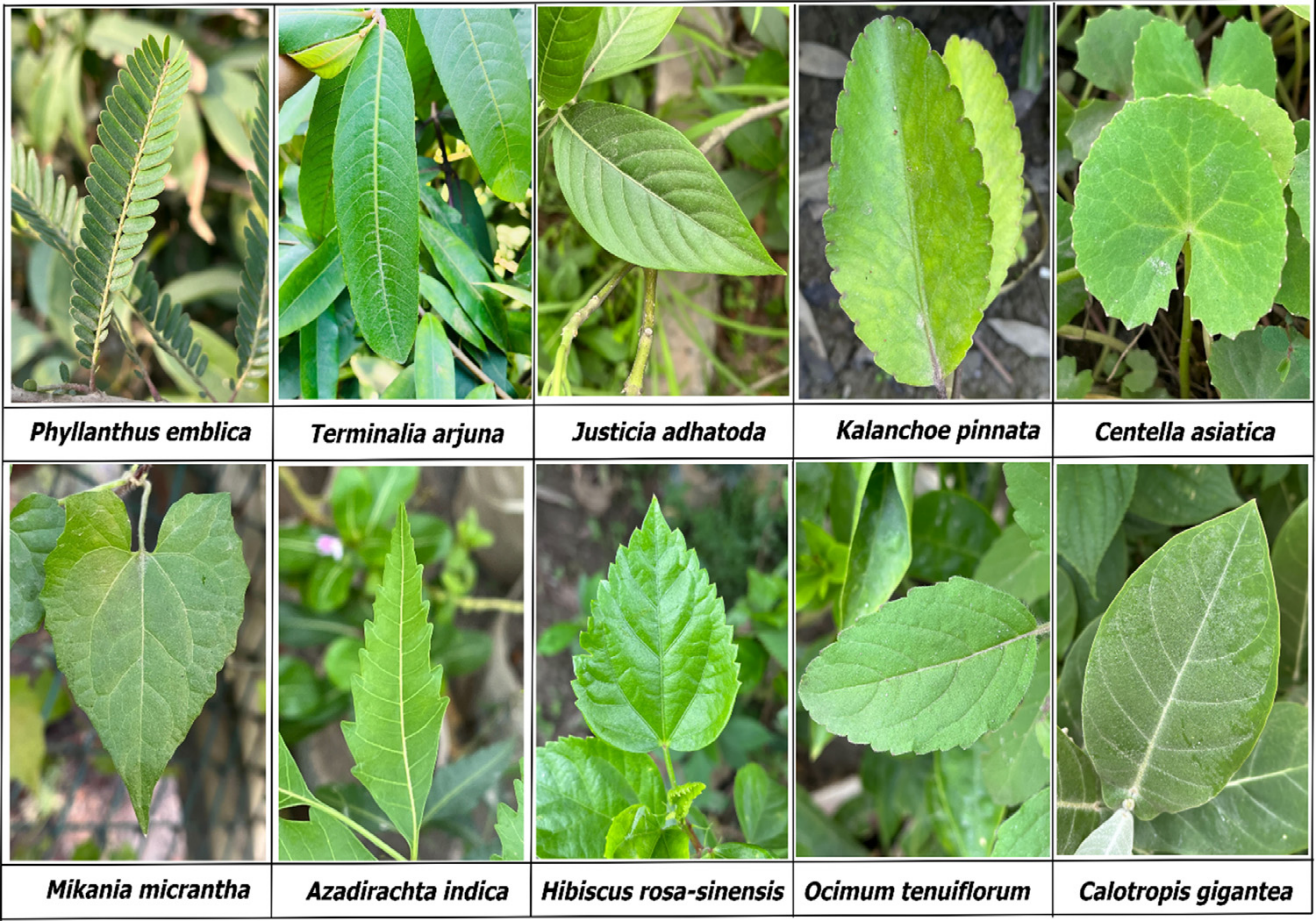
Sample images from each class of the BDMediLeaves dataset.

The following are the names of the ten medicinal plant species included in the “BDMediLeaves” dataset, along with a brief discussion about their medicinal benefits:

***Phyllanthus emblica*:***Phyllanthus emblica,* commonly known as “Amalaki” in Bengali, has been widely utilized in Ayurveda for both its medicinal properties and as an edible (tonic) herb. It is highly nutritious and serves as a valuable and healthful source of essential nutrients such as vitamin C, minerals, and amino acids [4]. Every component of this plant is harnessed for its therapeutic potential, specifically fruits which contain oil, vitamins, amino acids, and fatty acids. In this dataset, there are 200 original images in this category.
***Terminalia arjuna*:***Terminalia arjuna,* usually known as “Arjun” in Bengali, has been traditionally used to cure a variety of ailments. The effectiveness of this plant as anti-inflammatory, anticoagulant, antihypertensive, antifungal, cardioprotective properties, and antibacterial medication has been well documented [3]. This category contains 214 original images in this dataset.
***Justicia adhatoda*:** In Ayurvedic and Unani medicine, *Justicia adhatoda*, typically known as “Basak” in Bengali, is a well-known medicinal plant. It is utilized as a primary ingredient in various allopathic medicines for treating respiratory conditions such as cough, cold, and asthma [5]. Additionally, its leaves, flowers, and roots are utilized in the preparation of diverse herbal remedies. In this dataset, there are 200 original images in this category.
***Kalanchoe pinnata*:***Kalanchoe pinnata* is usually known as “Patharkuchi” in Bengali. It is also known as a wonder plant throughout the world. Apart from its high wound-healing properties, it is also well known for pharmacological qualities like antioxidant, antimicrobial, antidiabetic, antiviral, antitumor, antiallergic, and antidepressant [6]. This category contains 229 original images in this dataset.
***Centella asiatica*:***Centella asiatica*, commonly known as “Thankuni” in Bengali, is a traditional Ayurvedic herb widely recognized in Asia for treating various ailments. The aerial parts and roots of this herb are utilized for medicinal purposes, and its chemical constituents offer a diverse range of therapeutic benefits, including antibacterial, anti-inflammatory, anti-cancer, neuroprotective, antioxidant, and wound healing properties [7]. In this dataset, there are 201 original images in this category.
***Mikania micrantha*:** Bangladesh is one of the tropical Asian nations that are home to the medicinal plant *Mikania micrantha*. The utilization of this plant as traditional medicine has been observed in various regions of the world, primarily due to its therapeutic, antimicrobial, and invasive properties. The aforementioned plant has been reported to be mostly utilized for the treatment of diverse ailments, such as respiratory diseases, skin infections, and inflammatory conditions [8]. This category contains 203 original images in this dataset.
***Azadirachta indica***: *Azadirachta indica*, also referred to as “Neem” in South Asian regions, is a botanical specimen renowned for its broad therapeutic attributes. Its notable anti-inflammatory, antiviral, antibacterial, and antifungal characteristics have rendered it a prominent element in traditional medicine. The efficacy of Neem has been reported in the treatment of a range of diseases, including skin disorders, and digestive diseases, and in the fight against periodontal pathogens and dental plaque-causing bacteria that are responsible for dental caries [9]. In this dataset, there are 205 original images in this category.
***Hibiscus rosa-sinensis***: The leaves of *Hibiscus rosa-sinensis*, commonly referred to as “Joba” in Bengali, have been utilized in traditional medicine for their analgesic, antipyretic, anti-asthmatic, and anti-inflammatory attributes, as well as their potential anti-carcinogenic properties. Several studies have also reported the presence of antioxidant, antifungal, and antimicrobial properties in the leaves and flowers of *Hibiscus rosa-sinensis* [10]. This category contains 215 original images in this dataset.
***Ocimum tenuiflorum***: *Ocimum tenuiflorum*, commonly known as “Tulsi” in Bengali, is a widely used medicinal plant in South Asia. The scientific literature extensively documents the bioactive components of *Ocimum tenuiflorum*, which are attributed to its various therapeutic properties including but not limited to antioxidant, antibacterial, antidiabetic, antifungal, hepatoprotective, anti-inflammatory, and anti-ulcer effects [11]. In this dataset, there are 151 original images in this category.
***Calotropis gigantea***: *Calotropis gigantea*, known as “Akanda” in Bengali, is a perennial herb, and has been widely reported to offer notable medicinal benefits. It possesses properties such as antimicrobial, antiparasitic, and wound-healing effects. Numerous disease prevention and control methods have made use of the diverse phytochemicals present in *Calotropis gigantea*. These include the treatment of conditions like leprosy, cancer, ulcers, and elephantiasis, highlighting the plant's therapeutic potential and the significance of compounds such as flavonoids, tannins, cardiac glycosides, and terpenoids found throughout the plant [12]. This category contains 211 original images in this dataset. Fig. 2, presents the number of original images in each class. In Fig. 3, we present some of the sample images of the dataset. Table 1 presents the number of training, validation, and test images from the original (non-augmented) images, and total number of augmented images from each class. The descriptive statistics of the original dataset revealed that, the mean value of the images in each category was calculated as 202.90, with a median of 204.00. The image count ranged from 151 to 229, and the standard deviation was 20.37. The quartile information was as follows: the 25^th^ percentile (Q1) was 200.00, the 50^th^ percentile (Q2) was 204.00, and the 75^th^ percentile (Q3) was 214.25.

**Table 1:** Number of images in each class of the proposed BDMediLeaves dataset.

	Number of original images				

Class name	Train	Validation	Test	Total	Number of augmented images
*Phyllanthus emblica*	140	40	20	200	3,863
*Terminalia arjuna*	150	43	21	214	3,841
*Justicia adhatoda*	140	40	20	200	3,863
*Kalanchoe pinnata*	160	46	23	229	3,837
*Centella asiatica*	141	40	20	201	3,881
*Mikania micrantha*	142	40	21	203	3,919
*Azadirachta indica*	144	41	20	205	3,957
*Hibiscus rosa-sinensis*	151	43	21	215	3,861
*Ocimum tenuiflorum*	121	30	15	151	3,789
*Calotropis gigantea*	169	43	21	211	3,795
**Total Images**				**2,029** **(original)**	**38,606** **(augmented)**

II. The proposed “BDMediLeaves” dataset is different from one of the recently published dataset presented by Borkatulla et al [13]. This dataset has six different medicinal leaf categories compared to the mentioned study in reference [13]. Moreover, images in our dataset are captured in diffused lighting and natural background enhances the precision of analysis and processing in comparison to the white backgrounds mentioned in reference [13]. The utilization of white backgrounds for images presents several challenges, including the occurrence of reflections, glare, color distortion, shadows, and complications in segmentation tasks.

It is essential to emphasize that while the aforementioned botanical species with medicinal properties are widely acknowledged for their manifold advantages, further scientific investigation and clinical experimentation are necessary to substantiate their effectiveness and to acquire insights for the eventual commercialization of their advantageous bioactive components.

## 4 Experimental Design, Materials and Methods

**Fig. 4:**
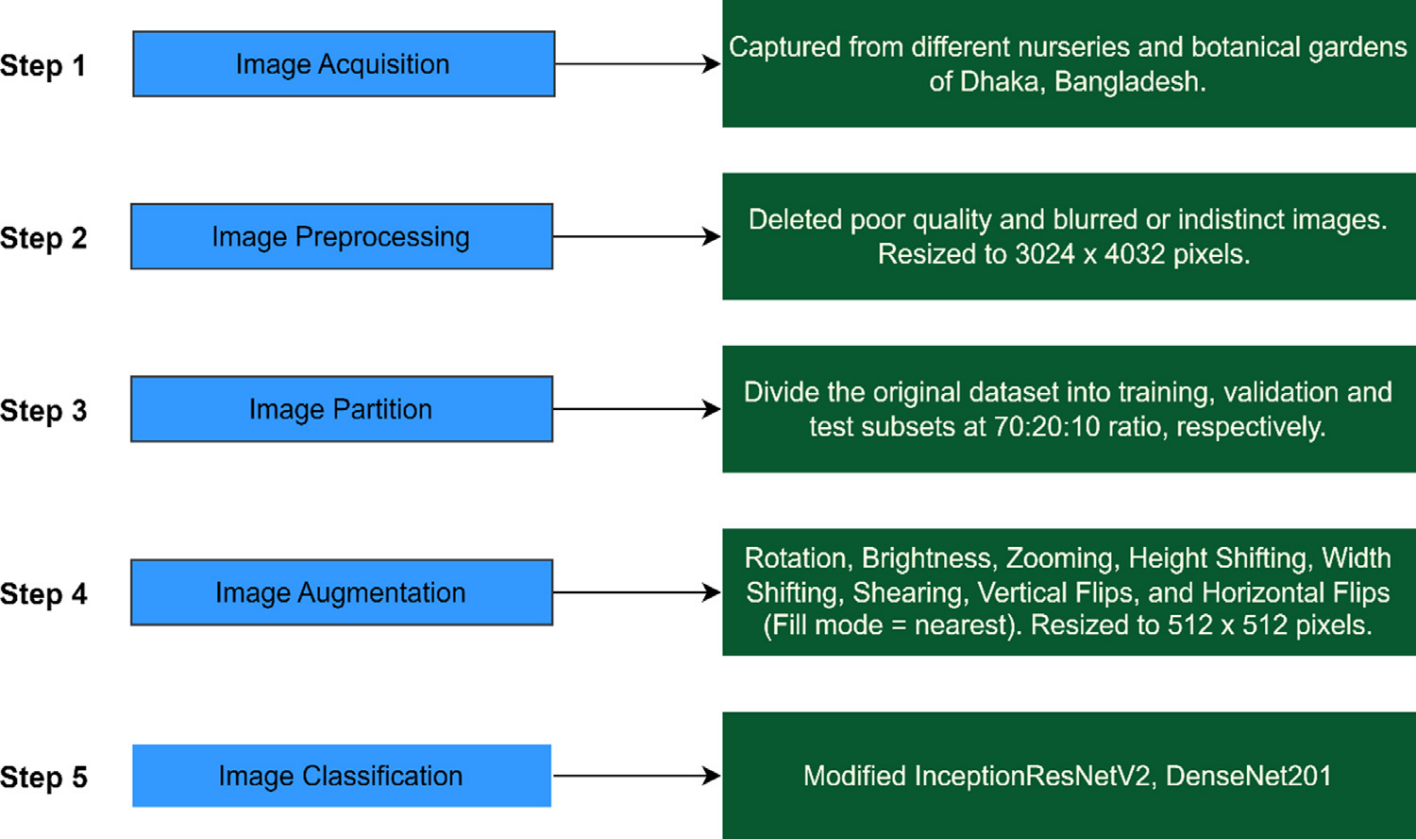
The development process of the BDMediLeaves dataset.

The development of the “BDMediLeaves” dataset consists of five steps: image acquisition, image preprocessing, image partitioning, image augmentation, and image classification (shown in Fig. 4). This section briefly describes each of these processes.

### 4.1 Image Acquisition System

The dataset consists of ten distinct classes of medicinal plants. The raw leaf images were captured from various botanical gardens and nurseries located in Dhaka, Bangladesh using iPhone 13 (with a 12 Megapixel camera, f/1.6, 26mm (wide), 1.7µm, dual pixel PDAF, sensor-shift OIS 12 MP, 13mm (ultra-wide), f/2.4, 120˚). A natural daylight background was maintained during the collection of all the images. To enhance leaf features and minimize shadows, we employed diffused lighting during the image acquisition process. We also maintained a consistent and natural background throughout the capture. To ensure consistent image quality, we conducted regular checks during the acquisition process. This involved verifying the focus, lighting, and capturing images from various angles to account for leaf variations. After collecting all the images, we performed quality control measures. Images with blurry areas, poor clarity, low contrast, or excessive brightness were identified and eliminated from the dataset. This particular step played a critical role in guaranteeing the inclusion of solely high-quality images for subsequent analysis. Through the elimination of these inferior images, we have ensured the integrity of the dataset and enhanced the reliability of subsequent analysis. A total of 2,310 leaf images were initially captured, from which a subset of 2,029 images was chosen to form the dataset proposed for this study.

### 4.2 Image Preprocessing

All the original images are of height (3024) x width (4032), Bit depth of 24, and horizontal and vertical resolution of 96 Dots per inch (DPI). Regarding the augmented images, the images underwent processing at a resolution of 512 × 512 pixels. The process of resizing the dimensions of the images was accomplished by employing the Image class from the Python Imaging Library, commonly referred to as PIL. The Image module offers a class called “Image” that is utilized to represent an image in the PIL. The module additionally offers various factory functions, encompassing the ability to import images from files and generate new images.

### 4.3 Image Partition

We provide a total of 2,029 original leaf images of ten different medicinal plants. To train and evaluate the ML and DL-based models, the original raw images were randomly divided into training, validation, and test subsets at 70:20:10 ratios for each category.

### 4.4 Image Augmentation

The process of image augmentation introduces diversity to images, thereby enhancing the overall generalizability and efficacy of ML and DL-based classification models [14]. To augment the number of images we have utilized the Keras ImageDataGenerator class. A variety of image augmentation techniques were employed using the aforementioned class, such as 60˚ rotation, 10% zooming, horizontal and vertical flips, brightness alteration (ranging from 0.2 to 0.3), 15% shearing, and 10% of height and width shifting, with the fill_mode property set to nearest. Image augmentation was applied to all the collected images before partitioning and placed in class-wise folders for researchers to work according to their needs. All the augmented images were resized to 512 × 512 pixels.

### 4.5 Medicinal Leaf/Plant Classification

**Fig. 5:**
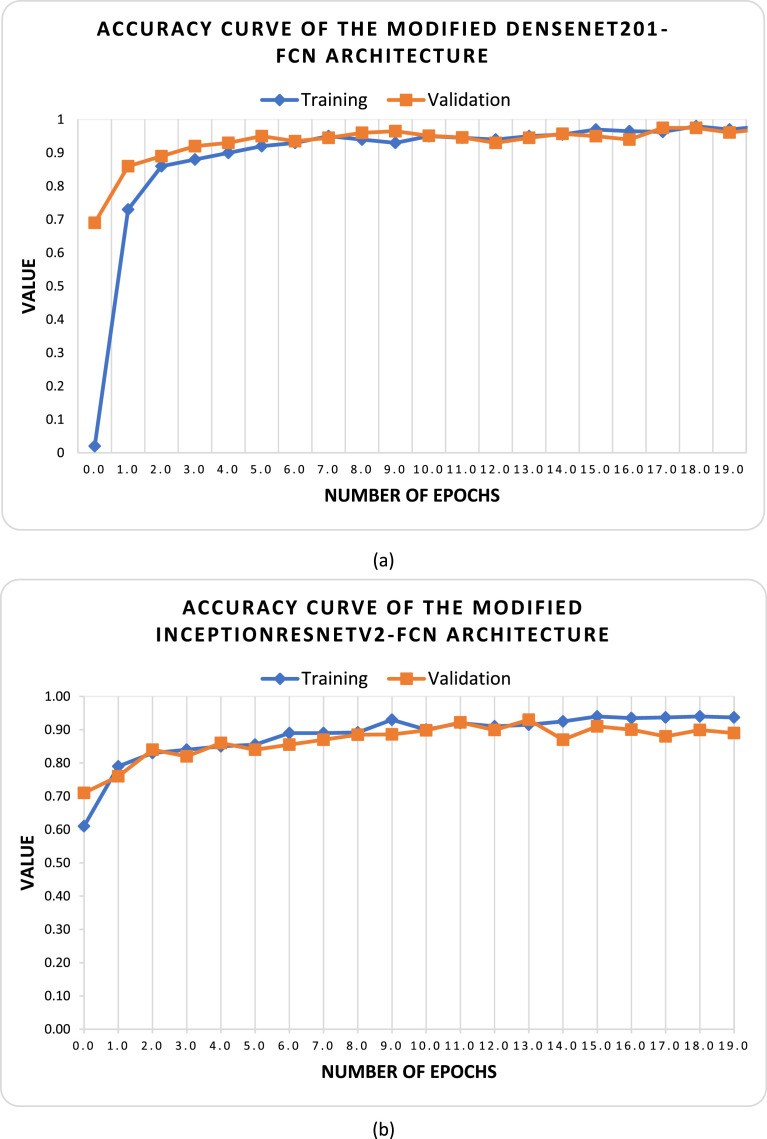

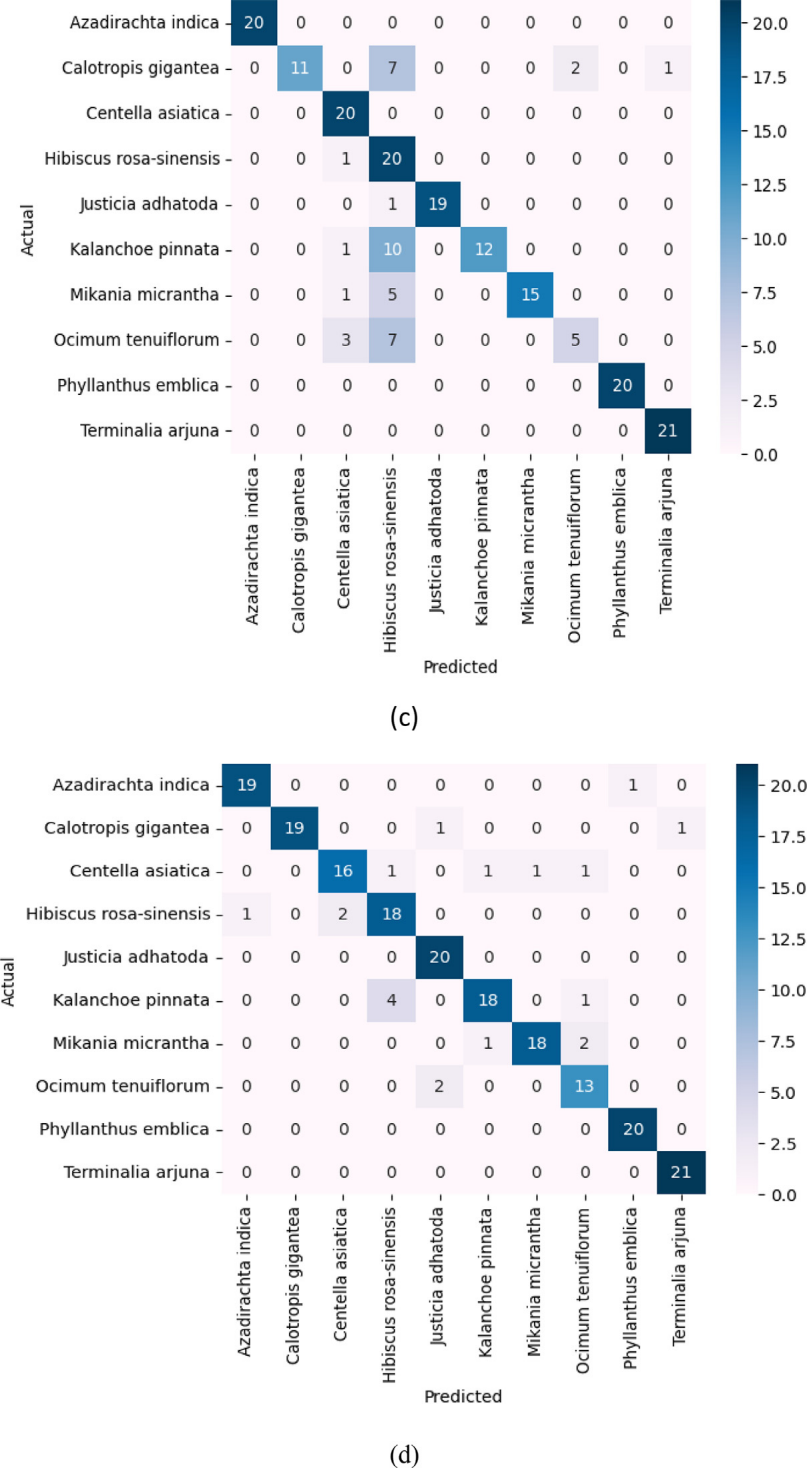
The training versus validation accuracy curve is shown for the proposed dataset using (a) the pre-trained DenseNet201-FCNs and (b) the pre-trained InceptionResNetV2-FCNs architectures. Additionally, (c) and (d) depict the confusion matrix evaluated on the unknown test dataset on the pre-trained DenseNet201-FCNs and pre-trained InceptionResNetV2-FCNs, respectively.

**Fig. 6:**
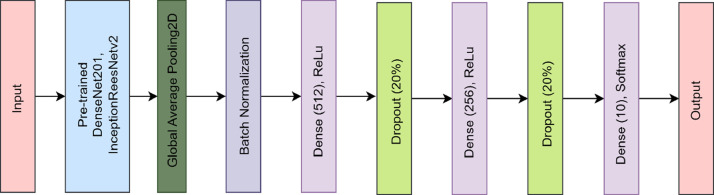
Network architectures on which the BDMediLeaves dataset is evaluated on.

To evaluate the performance of the proposed “BDMediLeaves” dataset, we have utilized two well-known Convolutional Neural Networks (CNN)-based DL models. These two CNN architectures are DenseNet201 [15] and InceptionResNetV2 [16]. DenseNet201 is specifically designed to address the challenge of vanishing gradients in neural networks. It also promotes the reuse of features across the network. This architecture provides dense connectivity, which establishes interconnections between each layer of the network in a feed-forward manner. InceptionResNetV2 is a widely recognized CNN. The InceptionResNetV2 model integrates the beneficial traits of the Inception and ResNet architectures. It utilizes the Inception modules to extract features at multiple scales and the residual connections to enhance gradient flow and network depth. All the images were resized to 224 × 224 × 3 to train the models. The pre-trained weights of ImageNet were preserved and all layers except for the fully connected layers were frozen for both architectures. Upon acquiring all the pre-trained features from both architectures, a global average pooling operation and batch normalization were implemented, subsequently followed by two fully connected networks (FCNs) consisting of 512, and 256 neurons, respectively. ReLU activation was applied in each of the dense layers, and a dropout of 20% neurons was added between the FCNs to mitigate the problem of overfitting in the models. In the ultimate dense layer, the quantity of neurons was set as ten, in accordance with the ten-class multiclass classification problem. The final FCN layer was implemented with the softmax activation function. Fig. 5 displays the training versus validation accuracy curves and confusion matrixes for the utilized architectures during the training process and testing process. In Fig. 5(a) and (b), the x-axis represents the number of training iterations or epochs, while the y-axis represents the accuracy performance metric for the pre-trained DenseNet201-FCNs and pre-trained InceptionResNetV2-FCNs architectures, respectively. Upon examining the accuracy curves for training and validation, it is evident that the proposed dataset provides ample instances for DL-based models to converge smoothly. The presented curves illustrate that the integrated DL-based models effectively converge with the provided image data during the initial, intermediate, and final phases of training. This demonstrates the proficient utilization of the proposed “BDMediLeaves” dataset in DL-based models for the task of medicinal leaf image classification. When assessing the convergence performance of the integrated models, it was observed that the pre-trained DenseNet201-FCNs architecture demonstrates faster convergence compared to the pre-trained InceptionResNetV2-FCNs architecture in both the training and validation stages of the proposed dataset. However, it is worth noting that in the test dataset, the pre-trained InceptionResNetV2-FCNs exhibited superior performance, with a lower number of misclassified instances of leaf images. This observation is supported by the confusion matrix provided in Fig. 5(c) and (d). The pre-trained DenseNet201-FCNs frequently misclassified the *Kalanchoe pinnata* category with *Hibiscus rosa-sinensis* category and *Ocimum tenuiflorum* category with *Hibiscus rosa-sinensis* category, as depicted in Fig. 5(c). Fig. 6 demonstrates a visual representation of the utilized network architectures. In Tables 2 and 3, we present a summary of the overall architecture of pre-trained DenseNet201-FCNs and pre-trained InceptionResNetV2-FCNs, respectively. These tables highlight the utilized layers, the output shape of each layer, and their corresponding parameter counts. Although the pre-trained DenseNet201-FCNs consume a larger number of trainable parameters, it converges faster compared to the pre-trained InceptionResNetV2-FCNs architecture. Table 4 shows the performance of the models on the augmented “BDMediLeaves” dataset. In the original test dataset portion, the pre-trained InceptionResNetV2-FCNs outperforms the pre-trained DenseNet201-FCNs architecture, although the latter converges better with higher validation scores in all the evaluation metrics.

**Table 2:** Pre-trained DenseNet201-FCNs architecture summary.

Layer (type)	Output shape	No. of parameters
input_1 (InputLayer)	[(None, 224, 224, 3)]	0
densenet201 (Functional)	(None, 7, 7, 1920)	18321984
global_average_pooling2d (GlobalAveragePooling2D)	(None, 1920)	0
batch_normalization (BatchNormalization)	(None, 1920)	7680
dense (Dense)	(None, 512)	983552
dropout_1 (Dropout)	(None, 512)	0
dense_1 (Dense)	(None, 256)	131328
dropout_2 (Dropout)	(None, 256)	0
dense_2 (Dense)	(None, 10)	2570

Total parameters: 19,447,114.

Trainable parameters: 1,121,290.

Non-trainable parameters: 18,325,824.

**Table 3:** Pre-trained InceptionResNetV2-FCNs architecture summary.

Layer (type)	Output Shape	No. of parameters
input_1 (InputLayer)	[(None, 224, 224, 3)]	0
inception_resnet_v2 (Functional)	(None, 5, 5, 1536)	54336736
global_average_pooling2d (GlobalAveragePooling2D)	(None, 1536)	0
batch_normalization_203 (BatchNormalization)	(None, 1536)	6144
dense (Dense)	(None, 512)	786944
dropout_1 (Dropout)	(None, 512)	0
dense_1 (Dense)	(None, 256)	131328
dropout_2 (Dropout)	(None, 256)	0
dense_2 (Dense)	(None, 10)	2570

Total parameters: 55,263,722.

Trainable parameters: 923,914.

Non-trainable parameters: 54,339,808.

**Table 4:** Performance of the pre-trained DenseNet201-FCNs and pre-trained InceptionResNetV2-FCNs architectures on the augmented dataset.

Model	Train Accuracy %	Validation Accuracy %	Test Accuracy %	Validation Precision %	Validation Recall %	Validation F1-Score %
Pre-trained DenseNet201-FCNs (224 × 224 × 3)	98.46	96.30	80.69	96.81	95.43	96.10
Pre-trained InceptionResNetV2-FCNs (224 × 224 × 3)	92.93	90.10	90.09	90.83	87.72	88.94

## Limitations

Not applicable.

## Ethics Statement

The research was carried out in strict accordance with ethical principles, demonstrating a commitment to the highest standards. Throughout the data collection process, no harm was inflicted upon any plants or animals. It is important to note that all images obtained were done so with the explicit consent of the respective owners, including those responsible for plants, gardens, nurseries, and similar entities. No written consent was needed to capture the images. The authors have read and follow the ethical requirements for publication in Data in Brief and confirming that the current work does not involve human subjects, animal experiments, or any data collected from social media platforms.

## CRediT Author Statement

**Saiful Islam:** Data curation, Formal analysis, Methodology, Visualization; **Md. Rayhan Ahmed:** Conceptualization, Supervision, Formal analysis, Software, Project administration, Investigation, Validation, Writing – original draft, Writing – review & editing; **Siful Islam:** Data curation, Research; **Md. Mahfuzul Alam Rishad:** Data curation, Formal analysis, Visualization; **Sayem Ahmed:**Methodology, Formal analysis; **Toyabur Rahman Utshow:** Methodology, Resources; **Minhajul Islam Siam:** Methodology, Resources.

## Declaration of Competing Interests

The authors declare that they have no known competing financial interests or personal relationships that could have appeared to influence the work reported in this paper.

## Data Availability

BDMediLeaves: A leaf images dataset for Bangladeshi medicinal plants identification (Original data) (Mendeley Data).

## References

[1] Ahmed M.R., Fahim M.A.I., Islam A.K.M.M., Islam S., Shatabda S. (2023). DOLG-NeXt: Convolutional neural network with deep orthogonal fusion of local and global features for biomedical image segmentation. Neurocomputing.

[2] Ahmed M.R. (2021). Leveraging convolutional neural network and transfer learning for cotton plant and leaf disease recognition. Int. J. Image Graph. Signal Process..

[3] Jain S., Yadav P.P., Gill V., Vasudeva N., Singla N. (2009). Terminalia arjuna a sacred medicinal plant: phytochemical and pharmacological profile. Phytochem. Rev..

[4] Gaire B.P., Subedi L. (2014). Phytochemistry, pharmacology and medicinal properties of Phyllanthus emblica Linn. Chin. J. Integr. Med..

[5] Dhankhar S., Kaur R., Ruhil S., Balhara M., Dhankhar S., Chhillar a K. (2011). A review on Justicia adhatoda: a potential source of natural medicine. Afr. J. Plant Sci..

[6] Rajsekhar P.B., Arvind Bharani R.S., Ramachandran M., Jini Angel K., Rajsekhar S.P.V. (2016). The ‘wonder plant’ Kalanchoe pinnata (linn.) pers.: a review. J. Appl. Pharm. Sci..

[7] Prakash V., Jaiswal N., Srivastava M. (2017). A review on medicinal properties of Centella asiatica. Asian J. Pharm. Clin. Res..

[8] Sheam M.M., Haque Z., Nain Z. (2020). Towards the antimicrobial, therapeutic and invasive properties of mikania micrantha knuth: a brief overview. J. Adv. Biotechnol. Exp. Ther..

[9] Lakshmi T., Krishnan V., Rajendran R., Madhusudhanan N. (2015). Azadirachta indica: a herbal panacea in dentistry - an update. Pharmacogn. Rev..

[10] Missoum A. (2018). An update review on Hibiscus rosa sinensis phytochemistry and medicinal uses. J. Ayurvedic Herb. Med..

[11] Cohen M.M. (2014). Tulsi - Ocimum sanctum: a herb for all reasons. J. Ayurveda Integr. Med..

[12] Raju P., Natarajan S. (2022). Investigation of pesticidal and anti-biofilm potential of Calotropis gigantea Latex Encapsulated Zeolitic Imidazole Nanoframeworks. J. Inorg. Organomet. Polym. Mater..

[13] Borkatulla B., Ferdous J., Uddin A.H., Mahmud P. (2023). Bangladeshi medicinal plant dataset. Data Br..

[14] Ahmed M.R., Islam S., Islam A.M., Shatabda S. (2023). An ensemble 1D-CNN-LSTM-GRU model with data augmentation for speech emotion recognition. Expert Syst. Appl..

[15] Huang G., Liu Z., Van Der Maaten L., Weinberger K.Q. (2017). Proceedings - 30th IEEE Conference on Computer Vision and Pattern Recognition, CVPR 2017.

[16] Szegedy C., Ioffe S., Vanhoucke V., Alemi A.A. (2017). 31st AAAI Conference on Artificial Intelligence, AAAI 2017.

